# Layered 3D Covalent Organic Framework Films Based on Carbon–Carbon Bonds

**DOI:** 10.1021/jacs.3c06621

**Published:** 2023-08-15

**Authors:** Yizhou Yang, Martin Ratsch, Austin M. Evans, Karl Börjesson

**Affiliations:** † Department of Chemistry and Molecular Biology, University of Gothenburg, 412 96 Göteborg, Sweden; ‡ George and Josephine Butler Polymer Laboratory, Department of Chemistry, University of Florida, Gainesville, Florida 32611-7200, United States

## Abstract

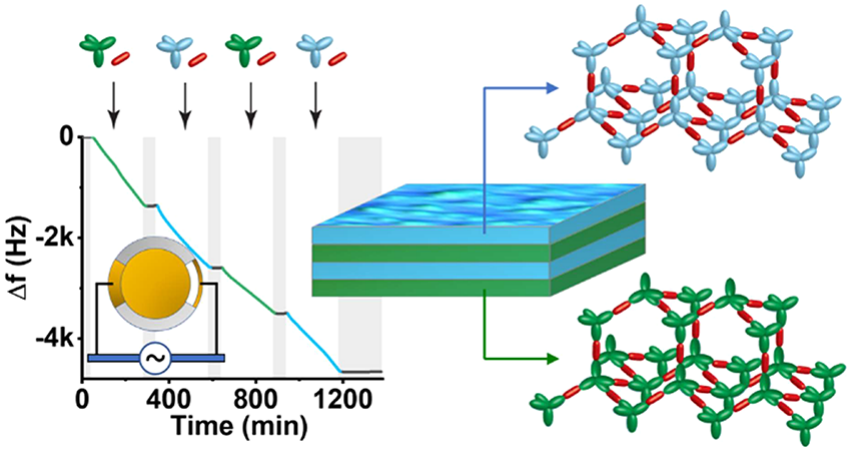

The development of covalent organic frameworks (COFs) during the past decades has led to a variety of promising applications within gas storage, catalysis, drug delivery, and sensing. Even though most described synthesis methods result in powdery COFs with uncontrolled grain size, several approaches to grow COF films have recently been explored. However, in all COFs so far presented, the isolated materials are chemically homogeneous, with all functionalities homogeneously distributed throughout the entire material. Strategies to synthetically manipulate the spatial distribution of functionalities in a single film would be game changing. Specifically, this would allow for the introduction of local functionalities and even consecutive functions in single frameworks, thus broadening their synthetic versatility and application potential. Here, we synthesize two 3D crystalline COF films. The frameworks, the ionic B-based and neutral C-based COFs, have similar unit cell parameters, which enables their epitaxial stacking in a layered 3D COF film. The film growth was monitored in real time using a quartz crystal microbalance, showing linear growth with respect to reaction time. The high degree of polymerization was confirmed by chemical analysis and vibrational spectroscopy. Their polycrystalline and anisotropic natures were confirmed with grazing incidence X-ray diffraction. We further expand the scope of the concept by making layered films from COF-300 and its iodinated derivative. Finally, the work presented here will pave the path for multifunctional COF films where concurrent functionalities are embedded in the same crystalline material.

## Introduction

Controlling the chemical structure of materials at the nano-, micro-, and macroscale is synthetically challenging. Still, it can be achieved by reticular chemistry, which is based on small building blocks that are linked together into stable crystalline lattices with a controlled nanoscale structure. By utilizing this strategy, several classes of materials have arisen—first the metal–organic framework (MOF)^1−7^ and later its siblings, the zeolitic imidazolate framework,^8−10^ and the covalent organic framework (COF).^11−14^ Per definition, these materials are homogeneous, which ensure consistent material properties, but at the same time the homogeneous nature limits the spatially directed incorporation of chemical functionalities. We will demonstrate here how functionalities can be embedded into defined regions of COF films by adding time-resolution control to reticular chemistry.

In COFs the small building blocks are linked together using covalent bonds, which results in a framework with considerable rigidity and stability. The majority of COFs are synthesized using a heterocoupling reaction,^11−15^ in which two building blocks with different functional groups react with each other. Alternatively, a few are grown using a homocoupling reaction,^16^ where two building blocks with the same functional groups react. COFs exist as 2D and 3D materials. 2D COFs have planar building blocks that polymerize into sheets that stack with the help of π–π interactions, forming anisotropic three-dimensional frameworks with laminar pores.^17−19^ Controlling the covalent in-plane and supramolecular cross-plane growth makes the directed synthesis of 2D COFs exceptionally challenging.^20−22^ A different approach to control multidimensional polymerization is to rely on an all-covalent 3D structure. These so-called 3D COFs are often based on one tetrahedral and one linear building block, which leads to open channels in all directions.^23−25^

The buildup of COFs and their potential applications within chemical separations,^26−28^ electroactive materials,^16,29−31^ water purification,^32^ catalysis,^14,33−35^ gas storage,^36−38^ and sensing^39,40^ have been intensely studied during the past two decades. Nevertheless, the controlled introduction of different functionalities within the same framework remains a challenge. The conceptual idea is simple: just like a skyscraper has apartments in some floors and offices in others, a controlled distribution of different functionalities within one framework would open a range of new possibilities to incorporate multiple and consecutive functions. The development of this idea of functional compartmentalization within MOFs is already quite sophisticated, as layered materials have been explored.^41−44^ However, they usually contain frameworks with different unit cells, which results in lattice mismatching and partial defects of the coordination bonds on the interlayer between the two frameworks. Further, there are only a few studied examples of COF–MOF composites^45−47^ and 2D COF layered materials,^48^ but all of these examples fail to generate a material that is regularly covalently linked, detracting from the advantages of reticular chemistry. To the best of our knowledge, the compartmentalization challenge remains unsolved for 3D COFs.

COFs are most often synthesized via a solvothermal approach, which results in highly crystalline COFs, but with uncontrolled grain size, making this method incompatible with layered structures.^12^ To enable COF films to be made, interfacial polymerization,^49−55^ mechanochemical synthesis,^56−58^ electrophoretic deposition,^59^ blade casting,^26^ and exfoliation and reassembly^60^ procedures have been used. While those techniques offer the possibility to grow frameworks with specified thickness, no examples of introducing compartmentalization into the framework have been observed. To catch up with the refinement of MOF technology and compartmentalize functions in COFs in the form of layers in a film, batch procedures seem not to be sufficient. This is because it is challenging to control the chemistry on the framework surface in situ. A solution for this problem is to add temporal control to the principles of covalent reticular chemistry. A continuous flow approach gives the possibility to change the building blocks reacting with a surface at any given time. We have previously shown that it is possible to grow all-carbon-linked porous aromatic frameworks (PAF-1 and BCMP-2) in a templated surface reaction in continuous flow (TSRCF)^61^ and further expand the TSRCF scope to conductive COFs.^16^ Real-time tracking of the reaction on the surface allowed nanometer-precise control of the film thickness. It further allowed assessment of the kinetics of the reaction, concluding that building blocks bound directly to the surface in an epitaxial manner.^16^ In this work, we show how an alternated reactant supply in continuous flow leads to a material containing alternating layers of two different 3D COFs. We thus provide a strategy to enable compartmentalization functions within COFs by adding a time dimension to reticular chemistry.

## Results and Discussion

**Figure 1:**
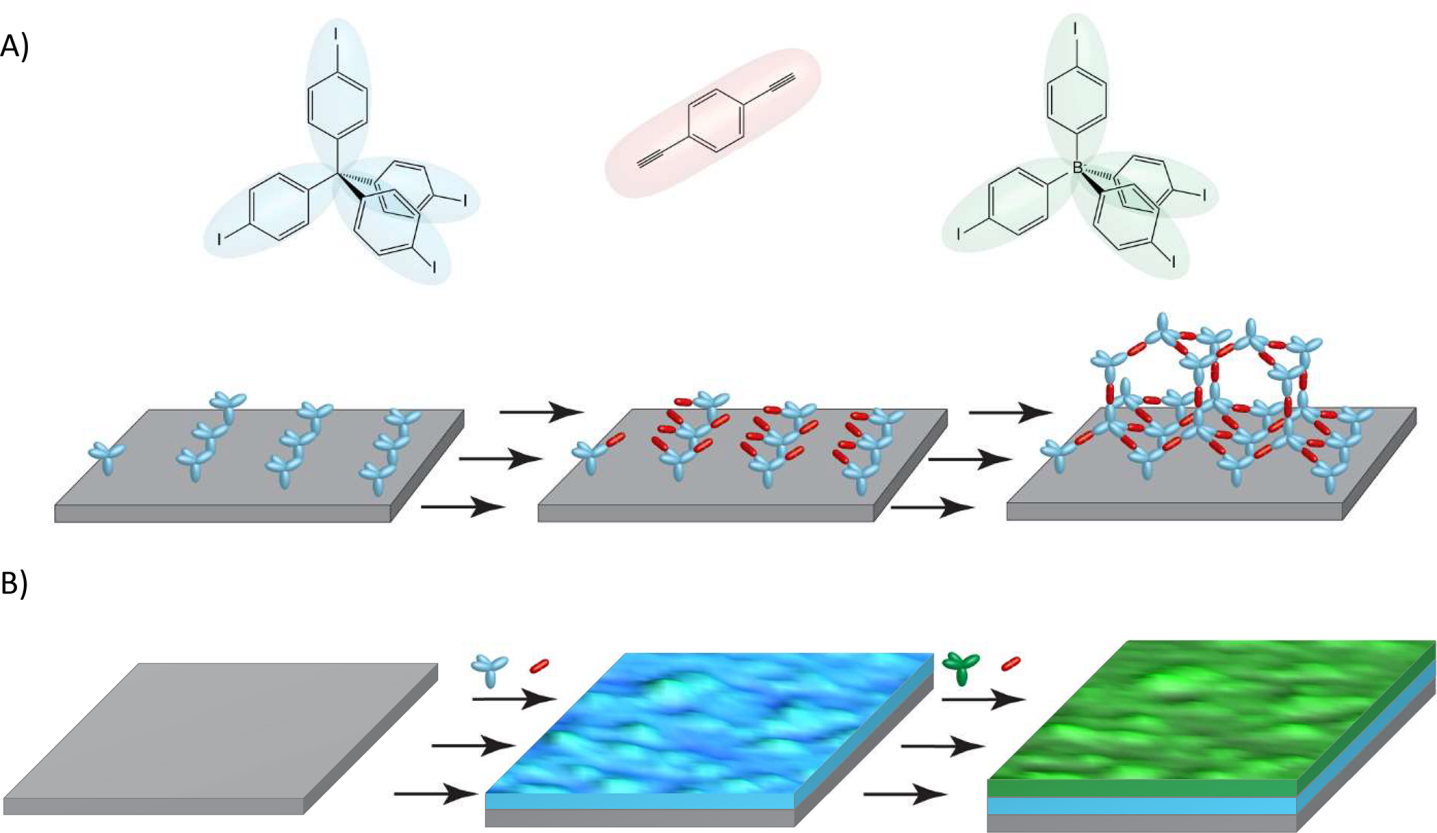
A templated surface reaction in continuous flow. (A) Chemical structure of TIPM (blue tetrapod), diethynylbenzene (red ellipse), and LTIPB (green tetrapod). Flowing only TIPM and diethynylbenzene together with the catalyst over the templated surface leads to the gradual buildup of a monolayered COF. (B) An alternating flow of two different building blocks such as TIPM and LTIPB together with diethynylbenzene and a catalyst result in a layered COF. Because the monolayers have the same unit cell parameters and the same functional groups, the layers (here blue and green) are connected via covalent bonds.

When different framework materials are grown on top of each other, they need to have the same geometry and lattice parameters in order to avoid tension at the interface. Additionally, the use of nonreversal linkage chemistry is required to prevent units in the framework to move in a diffusive kind of way with time. As a test bed for constructing layered 3D COFs, the building blocks tetrakis(4-iodoophenyl)methane (TIPM) and lithium tetrakis(4-iodophenyl)borate (LTIPB) were used (Figure 1 and Figure S1). Both have the same tetrahedral geometry, with the central atom (either C or B) being the only difference between them. The differences in C–C and B–C bond lengths are small (about 0.1 Å), and the lattice parameters of the boron– and carbon–COF made from these two materials are therefore expected to be relatively similar. Both building blocks can participate in Sonogashira heterocoupling reactions together with diethynylbenzene in order to form nondynamical C–C bonds. The reactions were set up in a continuous flow scheme in order to make films (Figure S2). By changing the flow input between TIPM and LTIPB, the framework lattice is not expected to change, but the boron atoms and the corresponding counterions exclusively appear at predefined depths in the film. Here, we will show that COF films can be made using both TIPM and LTIPB and that the similar XRD pattern from such films indicates a similar structure and thus lattice compatibility. Then layered COF films having a retained structure were built based on them. Toward the end of this article, we will expand the scope of the concept by making layered structures based on COF-300 and its iodinated derivative. The inclusion of iodine increases the level of electron scattering, allowing enough contrast for the individual layers to be visualized by SEM.

**Figure 2:**
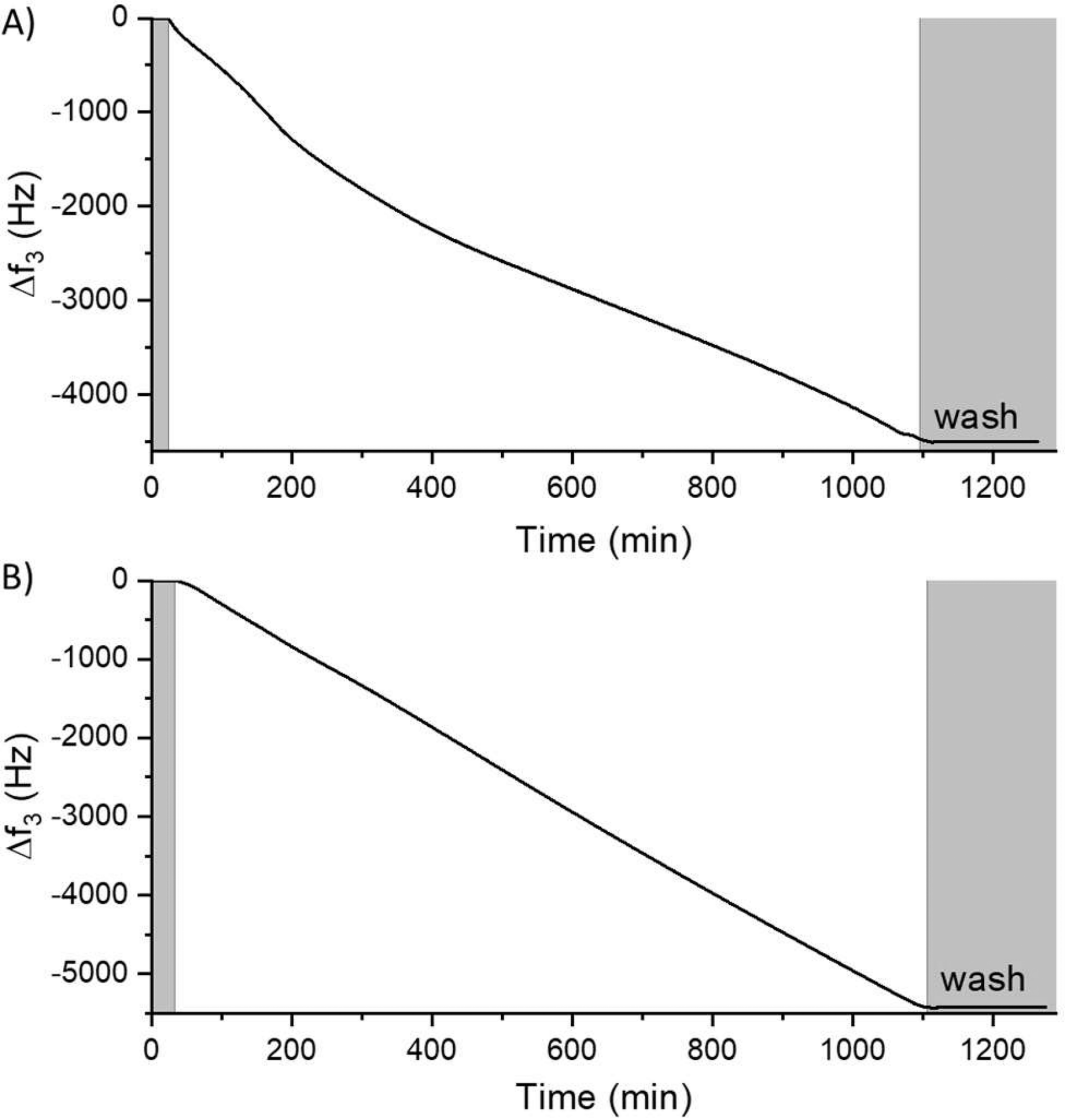
Measured resonance frequency decrease of the quartz crystal. Frequency change during the formation of (A) a boron–COF film and (B) a carbon–COF film. See the Methods section for reaction conditions.

We have previously developed technology to grow frameworks on self-assembled monolayer (SAM) templated gold surfaces and used a quartz crystal microbalance (QCM) to monitor the reaction progress.^16,61^ The measured parameter here is the resonance frequency shift of the quartz crystal induced by mass adsorption on the surface of the sensor. The continuous flow system makes it possible to change parameters such as the concentration, type of building block, and flow speed as often as desired during the reaction progress (Figure 1). It also has the advantage that it constantly transports new monomers to the surface, keeping the building block concentration constant, while all molecules that do not bind to the surface of the framework get washed out. Figure 2 shows the QCM frequency (and thus the deposited mass) as a function of time. The used reaction conditions and concentrations are the same for both building blocks, TIPM and LTIPB. The reaction rate on the surface is proportional to the derivative of the curve, and the monotonous slope indicates a steady reaction rate, which is as expected. We note that the reaction rate for TIPM is steadier than for LTIPB, which is a reproducible observation occurring at short time scales into the reaction progress, and we speculate that it is due to the ionic nature of LTIPB. The QCM frequency to thickness relationships of the films were determined by examining the depth of a deliberately made scratch using a profilometer. This resulted in a correlation of 0.04 nm Hz^–1^ for the boron– and carbon–COF films.

After confirming that the film growth on the templated gold surface is constant with time, we explored the kinetics of the reaction in more detail (Figure S3). The alkyne concentration does not influence the reaction rate significantly, indicating that processes involving TIPM in the catalytic cycle are rate-limiting for the overall reaction. In the absence of the aromatic halogen, tendencies of a Glaser–Hay coupling between two alkynes could be observed.^62^ The effect of this side reaction on the buildup of the framework was, however, deemed negligible, as this side reaction was suppressed in the presence of TIPM. Furthermore, to rule out any effect of the Glaser reaction on the structure of the framework, Cu-free Sonogashira coupling conditions was applied (Figures S4 and S5). These conditions produced similar films as the more standard Sonogashira coupling conditions, giving further support that the Glaser reaction does not influence the buildup of the framework material. However, the same solvent could not be used when coupling TIPM and LTIPB using Cu-free conditions, which causes monitoring issues when making layered structures, and the Cu-containing coupling conditions were therefore selected for future experiments.

**Figure 3:**
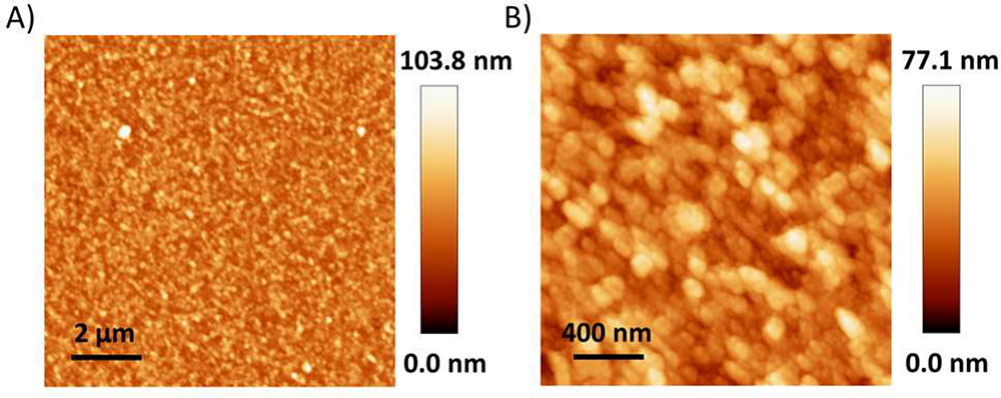
AFM analysis of a carbon–COF film. AFM height image of a large surface area (A) and a zoom-in area (B).

It has previously been shown that a lower concentration of the building blocks in TSRCF synthesis leads to increased film smoothness due to the different reaction orders in the bulk solution compared to on the surface.^16,61^ Atomic force microscopy (AFM) was therefore used to gain information about the surface morphology of produced films. Figure 3 and Figure S6 display AFM images of a carbon–COF and a boron–COF film, respectively. Both films show a uniform morphology over the scanned areas. The average roughness (*R*_a_) is 10.8 nm for a 55 nm thick carbon–COF film and 12.9 nm for a 110 nm thick boron–COF film when statistically estimated over an area of 4 μm^2^. The surface roughness is comparable to previously made BCMP-2 films, made by using similar concentrations.^54^ The average surface roughness sets a lower boundary for the thickness of the continuous thin films. Therefore, when making layered structures (vide infra), it is of importance that each layer is considerably thicker than about 10 nm, in order to be sure that layers are continuous and not intermixed.

**Figure 4:**
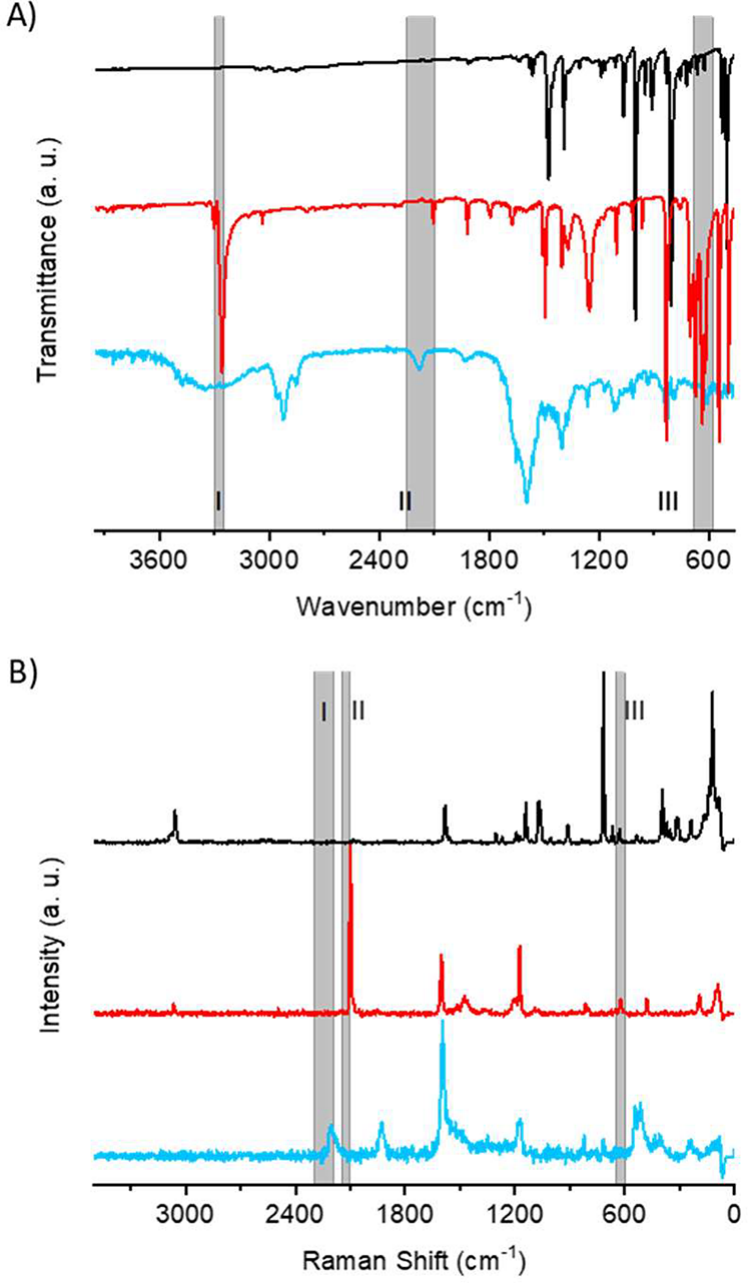
FT-IR and Raman spectra of a carbon–COF film compared to respective building blocks, indicating a high degree of polymerization. (A) FT-IR (an ATR accessory were used when measuring on the monomers) and (B) Raman spectra where the black (TIPM), red (diethynylbenzene), and blue (carbon–COF film) lines are compared. Characteristic signals to distinguish between disubstituted and terminal alkynes are located in the three gray regions (I–III).

In order to investigate the chemical uniformity of the framework, we examined the occurrence of unreacted triple bonds in the synthesized COF films. Fourier transform infrared (FT-IR) and Raman spectroscopy can distinguish between terminal and disubstituted alkynes and were therefore employed. The gray areas I–III in Figure 4 and Figure S7 are regions with characteristic signals related to carbon–carbon triple bonds. Area I in Figure 4A marks the absorption region for the stretch vibration between the terminal proton and adjacent carbon of a monosubstituted alkyne (≡C–H). Diethynylbenzene has a strong signal at 3260 cm^–1^,^63^ whereas no signal for the COF film is seen in this area. Area II represents the expected range for the C≡C stretching vibration of a disubstituted alkyne. In this area a prominent signal at 2180 cm^–1^ is seen for the COF film, but not in the spectra of the building blocks. Area III marks the region for the −C≡C–H bend vibration. Diethynylbenzene shows two peaks in this region, at 620 and 636 cm^–1^,^63^ whereas no signal is evident for the COF film. In summary, within the signal-to-noise ratio of the FT-IR spectra presented here, no signs of unreacted terminal alkynes can be seen. Instead, clear evidence of the formed disubstituted alkyne is evident, which together with the lack of terminal alkyne protons in our COF films indicates a high degree of polymerization. Furthermore, for comparison the FT-IR spectra of LTIPB and a boron–COF film were measured (Figure S7). A similar peak distribution was observed in these spectra, giving the conclusion of a high degree of polymerization here as well.

Raman spectroscopy was used to corroborate the FT-IR spectroscopy findings (Figure 4B and Figure S8). Area I marks the expected region for C≡C stretch vibrations of disubstituted alkynes.^64^ A signal at 2203 cm^–1^ in the carbon–COF film is clearly present, but no signal is evident in the spectra of the building blocks. Area II, between 2140 and 2100 cm^–1^, marks the expected region of C≡C stretch vibrations of terminal alkynes.^64^ A strong peak at 2104 cm^–1^ in this region is evident for diethynylbenzene,^65^ whereas no signal is observed for the carbon–COF film. Area III marks the expected region for −C≡C–H bend vibrations.^64^ A weak peak in this region is present for diethynylbenzene at 620 cm^–1^, but no signal exists for the carbon–COF film. It should be noted that the expected signal for the vibration between the terminal proton and the adjacent carbon of the terminal alkyne (≡C–H) is not visible, indicating that this vibration is not Raman active in our system. The analysis of the Raman spectra of LTIPB and the boron–COF film was performed (Figure S8), showing similar peak distributions. These vibrational spectroscopies demonstrate that a high degree of polymerization is observed in our temporally controlled syntheses.

If no terminal triple bonds are left in the film, then no iodine should be expected. In order to verify the lack of iodine in the COF films, XPS were performed (Figure S9 and Table S1). The obtained results confirm the expectation of a very low iodine content in the films. In summary, the findings from FT-IR, Raman, and XPS give a coherent picture of a high degree of polymerization in the as-obtained films.

**Figure 5:**
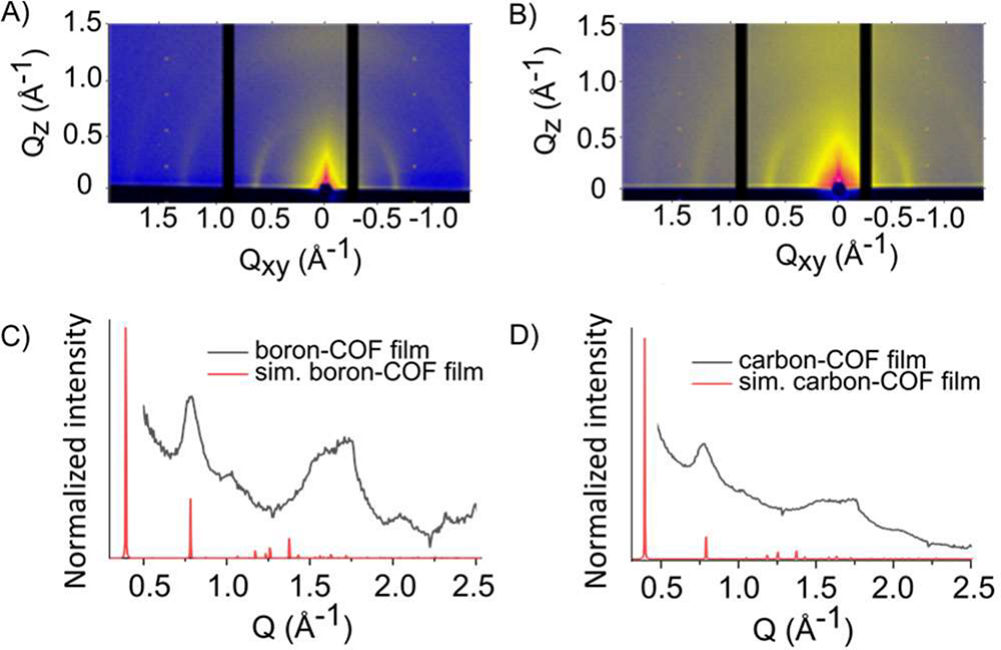
Measured and calculated grazing incidence X-ray diffraction patterns (GIXRD) for single-layered boron– and carbon–COFs. (A) GIXRD of the boron–COF film and (B) GIXRD of the carbon–COF film. (C) Comparison between the simulated boron–COF diffraction signals (red) with the measured spectrum (black). (D) Comparison between the simulated carbon–COF diffraction signals (red) with the measured spectrum (black).

The boron– and carbon–COF films have a high degree of polymerization, and if crystalline, these two materials are expected to have similar unit cells and thus diffraction patterns. In order to investigate the crystallinity of the two materials, grazing-incidence wide-angle X-ray scattering (GIXRD) was employed, and its result was compared with a computed non-interpenetrated model, which showed good agreement with the experimental X-ray pattern. The 2D grazing incidence diffraction patterns reveal that the boron– and carbon–COF films are isolated as polycrystalline conformal coatings with preferential orientations (Figure 5). In both films, the same peak locations were observed, and these were further consistent with a carbon–COF film constructed from tetrakis(4-bromophenyl)methane instead of TIPM (Figure S10). These observations led us to compare the experimental diffraction patterns to those simulated from the 3D network. The peak position found at 0.78 Å^–1^ agrees well with the simulated diffraction pattern from a diamondoid network with a unit cell dimension of 15.2 Å for both the boron– and carbon–COF films (Figure 5 and Figure S11). Consequently, both frameworks have similar lattice parameters (Figure 5 and Figure S12). Notably, the peak that is seen at 0.41 Å^–1^ in the simulations is not experimentally observable due to the limitations of observing low-*Q* scattering features in our grazing incidence diffraction instrument. Because of the limited amount of scattering information included in the experimental patterns, especially at low scattering angles, it is also challenging to assign the degree of interpenetration in these networks. Nonetheless, clear diffraction signals indicate that both networks are formed as crystalline materials with similar lattice structures.

The intensity of the diffracted beam along the half circle-like shape increases by around 25% toward *Q*_*Z*_ = 0 Å^–1^ (horizon) (Figure S13). This increase indicates a slightly preferred orientation of the framework. Collectively, these observations reveal that the films isolated are homostructural, polycrystalline, and oriented preferentially as anisotropic layers. Furthermore, both experimental diffraction data and modeling suggest the same lattice parameters for the C- and B-based films. Thus, these two materials are chemically and structurally compatible for the creation of heterostructured multilayered crystalline films.

**Figure 6:**
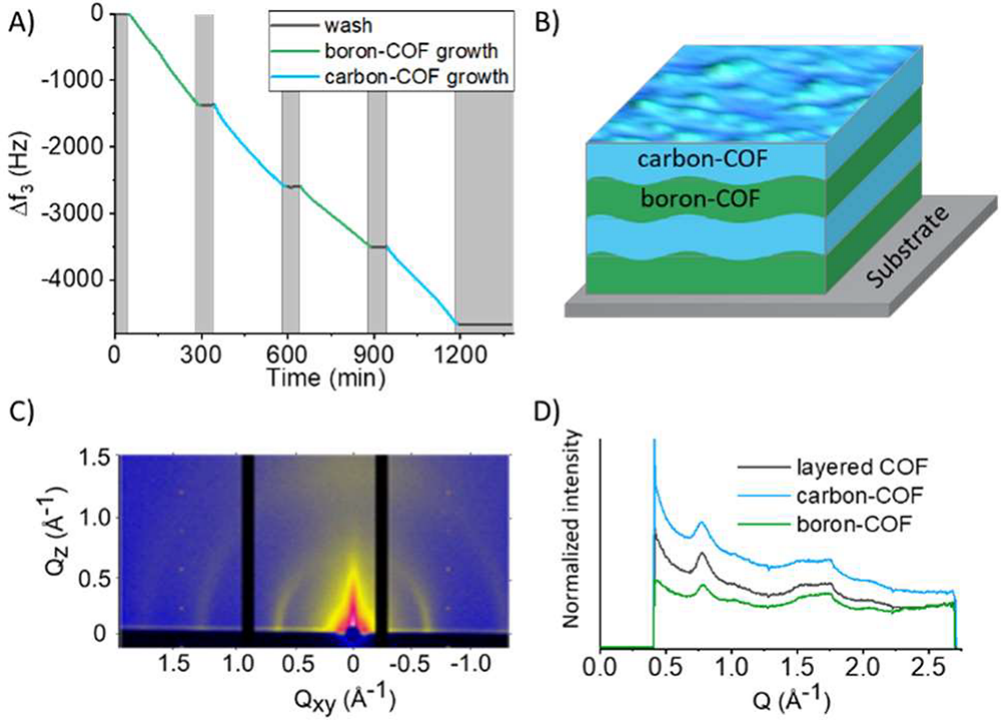
Layered COF film. (A) Resonance frequency decrease during the growth of a layered film and (B) schematic illustration of a layered film. The diagram under (A) shows the decrease of the resonance frequency (third overtone) during the formation of the four-layer film. Each layer was grown for 4 h at 25 °C followed by a washing period with only solvents for 1 h. (C) 2D GIXRD diffraction data of the layered COF and (D) the comparison of the 1D data of the layered films with its individual components (a carbon– and a boron–COF film). All three films show very similar diffraction patterns.

At this point, smooth films of framework materials having either C or B as central atoms can be made. Furthermore, these two materials have compatible lattice parameters, possibly enabling growth on top of each other. In the flow setup, the building blocks TIPM and LTIPB can be alternated while keeping the catalyst and alkyne running over the surface (Figure 1 and Figure S2). In such way the buildup of the B and C frameworks can be monitored in real time on the same surface. The result of such an experiment is shown in Figure 6, where the green line represents the QCM frequency at times where LTIPB flows over the surface and thus the buildup of the boron–COF film. The blue line represents times were TIPM flows over the surface and thus the buildup of the carbon–COF film. The black line represents times when only solvent flows over the surface. The QCM frequency shift shows the expected staircase shape during the growth of a four-layer film. Using the determined frequency to thickness relationship of 0.04 nm Hz^–1^, each layer has a thickness of 35–55 nm, which is larger than the average surface roughness. Hence, the individual layers are expected to be continuous. While flowing LTIPB or TIPM (both together with the alkyne and catalyst), a linear increase of mass with time was seen, and no change of mass is seen during washing. To further determine the thickness of each layer with accuracy, the thickness of the first layer and the final film were measured by AFM to 52 and 203 nm, respectively (Figure S14). The measured values are in good agreement with estimation from QCM data, illustrating a film density that was not changed during the sequential film growth process. Combining the measured thickness of 52 nm for the first layer with the modeled size of a unit cage (Figure S15), each layer corresponds to the buildup of about nine such unit cages. Also, the average frequency shift for each layer is about 1200 Hz, corresponding to a mass accumulation of 1800 ng/cm^2^ according to the Sauerbrey equation. By dividing the accumulated mass by the film thickness, we calculated the density of the film to be 0.34 g/cm^3^.

It is reported that films made in a continuous flow approach but not covalently connected to the underlying surface have a tendency to crack.^54^ No tendency of cracks could be observed in the layered films while investigating the surface with an optical microscope or SEM (Figure S16), supporting the conclusion that the frameworks are connected through covalent bonds when grown on top of each other. The surface morphology (Figure S17) and chemical environment (Figure S18) are also similar to those of the single components.

To confirm that the layered framework has the same lattice structure and crystallinity as monocomponent films, GIXRD was performed (Figure 6C). Clear diffraction patterns are present for a layered film, indicating a polycrystalline state. Comparing an overlay of the 1D projections of the scattering patterns for the layered film and the single-component films (Figure 6D), it is evident that the framework geometry in all three cases is the same. Thus, the lattice parameters and unit cells of those materials are equal, indicating that it is possible to epitaxially grow the frameworks on each other. In other words, that it is possible to build a film with layers of chemically distinguished 3D COFs on top of each other.

To expand the scope of the templated reaction in continuous flow, the developed protocol was also applied in Schiff-base chemistry to prepare layered imine-type COF films (Scheme S3). Furthermore, the use of Schiff-base chemistry is compatible with halogens, allowing iodine-modified monomers to be used. Thus, this enabled large contrast in electron scattering-based experiments between layers consisting of non-iodinated and iodinated monomers. By alternating the supply of tetrakis(4-aminophenyl)methane with either terephthalaldehyde or 2,5-diiodoterephthalaldehyde, we can control the COF-300 and COF-I-300 layers to realize periodical growth. As a first step, the thinness limit was explored. Layered films with ultralow thickness were made, showing a linear increase in mass addition with time (Figure S19A). The thicknesses of each layer in the films are smaller than the unit cell of COF-300 (Figure S19B). It is therefore not suitable to view such films as two different crystalline materials bound to each other, but rather as a single intermixed material. However, this experiment gives a demonstration of the very high sensitivity of the QCM and the superb thickness control when making surface reactions in continuous flow.

**Figure 7:**
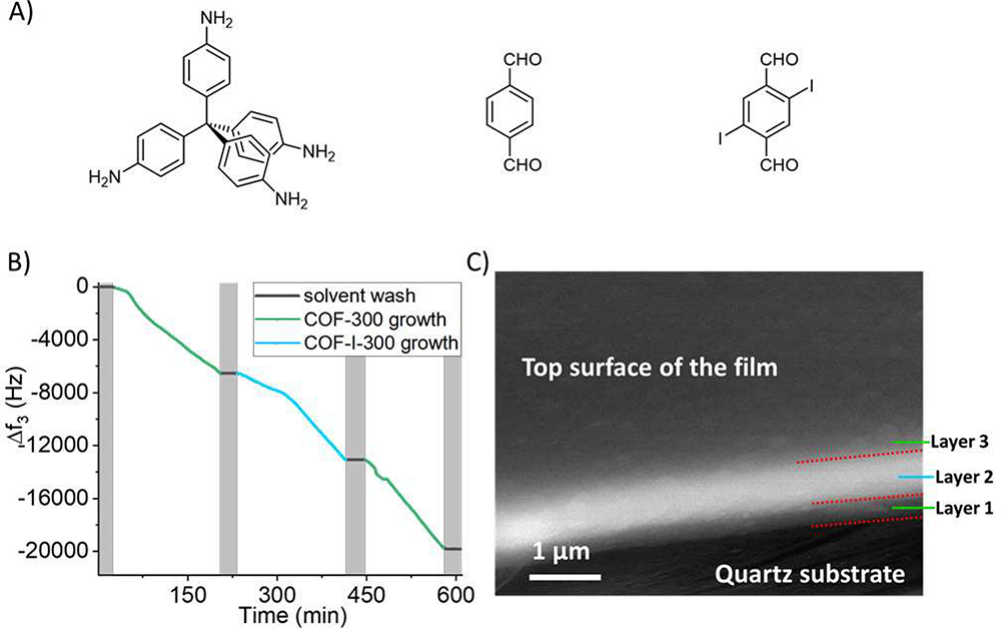
Layered imine-type COF film. (A) Chemical structures of tetrakis(4-aminophenyl)methane (left), terephthalaldehyde (middle), and 2,5-diiodoterephthalaldehyde (right), which were used to synthesize COF-300 and COF-I-300. (B) Resonance frequency decrease during the growth of a thick layered film with a configuration of A/B/A. (C) SEM image of the fractured film showing the layered fringe. The middle layer is relatively brighter compared to layers 1 and 3 due to the presence of the heavy atom iodine.

To directly observe the internal layered structure, a thick trilayered film with the configuration COF-300/COF-I-300/COF-300 was prepared (Figure 7B). The QCM chip was then broken so that the fractured film could be observed under SEM (Figure 7C and Figure S20). Because of the magnitude of electron scattering from an element being highly dependent on its atomic number, COF-I-300 is expected to give a much larger signal as compared to COF-300. Indeed, an adequate contrast can be seen between the layers at the section of the fractured film, clearly showing the layered structure. The film in Figure 7C was slightly hanging out from the support and was viewed at a small tilt angle. This allowed the Au layer in between the quartz substrate and film to be covered by the film. The bottom part of Figure 7C therefore displays the scattering from quartz, followed by the bottom COF-300 layer, which has clearly defined boundaries both downward and upward. The next layer contains the highly scattering COF-I-300, also with clearly defined lower and upper boundaries. The top COF-300 layer has a clearly defined boundary downward, but less so upward. This because of the tilted viewing angle, allowing both the fractured edge as well as the top area of the film to be viewed with a very small difference in contrast.

## Conclusions

Using a templated surface reaction in continuous flow, we have successfully constructed two all C–C-linked 3D COF films, having either a B or C atom as the central atom. The kinetics of the film buildup was monitored in real time using QCM. Both frameworks have a high degree of polymerization, as confirmed using XPS, FT-IR, and Raman spectroscopy. They have a low surface roughness, and their polycrystalline nature was confirmed using GIXRD. Because of their low surface roughness and similar lattice parameters, we were able to synthesize a layered 3D COF, consisting of B and C–COF layers covalently attached to another. We further expanded the scope of the concept to COF-300, where we used iodinated and non-iodinated building blocks to build up layered films.

To summarize, a controlled compartmentalization within a single COF film was achieved. Here, B or I was used as a proof-of-concept functionality, but we suggest that the presented strategy can be used to construct COF films with any local distribution of any functionality—this as long as the different COFs are topologically homologous and the introduced functionalities are compatible with the synthesis methodologies used in the COF synthesis. This work therefore paves the way for multifunctional COFs, where different and/or consecutive functions are embedded in the same crystalline material.

## Supporting Information Available

Materials and methods, synthesis details and characterization of building blocks including ^1^H NMR, synthesis details of films including schematics of the setup used, additional QCM, GIXRD, AFM, FT-IR, Raman, XPS, SEM data, and the simulated structure of the carbon–COF (PDF)
