# Supplementary material for: Layered 3D Covalent Organic Framework Films Based on Carbon–Carbon Bonds
Source: J Am Chem Soc. 2023 Aug 15;145(33):18668–75. doi: 10.1021/jacs.3c06621 (PMC10450803; doi:10.1021/jacs.3c06621)
Supplement: Supplementary file 1 — ja3c06621_si_001.pdf [file ja3c06621_si_001.pdf]

Supporting Information

Layered 3D Covalent Organic Framework Films Based on Carbon-carbon Bonds

Yizhou Yang[1], Martin Ratsch[1], Austin M. Evans[2], Karl Börjesson[1*]

[1] Department of Chemistry and Molecular Biology, University of Gothenburg, 412 96 Göteborg, Sweden

[2] Department of Chemistry, University of Florida, Gainesville, Florida 32611-7200, USA

Table of Contents

Materials and methods 2

Building block synthesis 3

Film-synthesis 4

Figures and tables 7

References 20

Materials and methods

General: All starting materials were purchased from Sigma-Aldrich Chemical Co. or TCI and used without further purification with the exception 1,4-diethynylbenzene, which was sublimated before use. All moisture and oxygen-sensitive reactions were carried out using Schlenk techniques in oven-dried glassware or in a nitrogen atmosphere glovebox. Solvents used for moisture and oxygen-sensitive reactions were dried using an MBraun MB SPS-800 solvent purification system. Flash chromatography was performed by a Teledyn Combiflash EZ prep using liquid chromatography mass spectrometry (LC-MS) grade solvents and normal phase silica with mesh size 230-400, particle size 40-63 micron, and pore size 60 Å. ^{1}H NMR spectra were recorded on a Varian spectrometer at 400 MHz. *J*-coupling values are given in Hertz (Hz) and chemical shifts are given in ppm using tetramethysilane (TMS; δ = 0.00 ppm) as an internal standard. The building block (lithium tetrakis(4-iodophenyl)borate) (LTIPB) was synthesized in one step starting from 1,4-diiodobenzene, following a literature procedure[1] that is described in detail in the building block synthesis. During the QCM-D measurements the frequency shifts and dissipation from the fundamental and 6 overtones (3rd, 5th, 7th, 9th, 11th and 13th) were recorded. For clarity, just the 3rd overtone for the frequency shift is presented.

COF films analysis: The X-ray diffraction was measured at a Mat:Nordic SAXS/WAXS/GISAXS, which is equipped with an Rigaku 003+ high brilliance microfocus Cu-radiation source and a Pilatus 300k/100k detector. X-ray photoelectron spectroscopy (XPS) was performed using a PHI5000 VersaProbe III – Scanning XPS Microprobe™ with a monochromatic AlKα X-ray (E = 1486.6 eV) X-ray source. The used beam size diameter was 100 µm at 25 W and 15 kV. AFM was performed using an NTEGRA AFM from NT-MDT Spectrum instruments. Raman spectroscopy was performed using an alpha300 R Raman microscope with a 532 nm laser line. Fourier transform infrared spectroscopy were performed on an Invenio R from Bruker. The building blocks TIPM and LTIPB were measured in an ATR set up with the detector being at ambivalent temperature. The COF films were measured with a reflection set up and a liquid nitrogen cooled detector (LN-MCT mid) in order to increase the signal to noise ratio. Simulations were conducted by first starting with reported single-crystal diamonoid 3D COF structures.[2] We then Pawley refined this structure to our experimental 1D X-ray diffraction pattern, which was obtained via integration of the 2D experimental pattern. Refinement was performed using the Reflex Module of MaterialsStudio. We find that this refinement is in good agreement with a diamonoid unit cell constructed from the monomers that were polymerized to produce this 3D framework. However, limited experimental scattering information, likely due to the limited thickness of these films, precluded more precise structural insight including the degree of interpenetration.

Building block synthesis

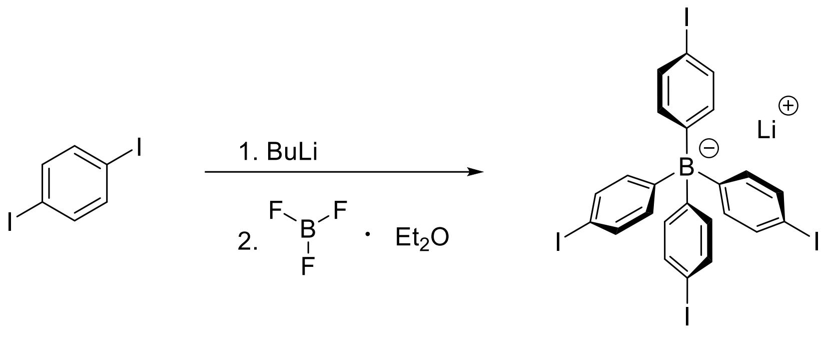

Scheme S1. Synthetic procedure of the boron building block LTIPB.

Synthesis of lithium tetrakis(4-iodophenyl)borate (LTIPB): A solution of 1,4-diiodobenzene (2.5 g, 7.58 mmol) in ether (75 mL) was stirred at -10 °C under N_2 atmosphere and treated dropwise with a solution of butyllithium (3.025 mL, 2.5 M in hexane, 7.5 mmol). The resulting mixture was kept at -10 °C for 10 min, and BF3·OEt$_2$ (0.199 g, 1.4 mmol) was then added. The temperature was allowed to rise to 25 °C, and after 18 h, the mixture was filtered to remove solids. The solid residue was washed with ether (50 mL), and the desired product was dissolved in acetone (5 mL). Acetone was afterwards removed from the filtered extracts by evaporation under reduced pressure, leaving a brown raisin like residue, which was stirred overnight in pentane. The resulting grey powder was recrystallized from DCM and LTIPB was received as a grey solid (0.73 g, 0.89 mmol, 63%). The proton NMR data matched literature values.[1]

^{1}H NMR (400 MHz, dmso-d6, r.t.): δ (ppm) = 7.29 (d, 8H, 3J = 8.4 Hz, ArH), 6.88 (m, 8H, ArH).

Film-synthesis

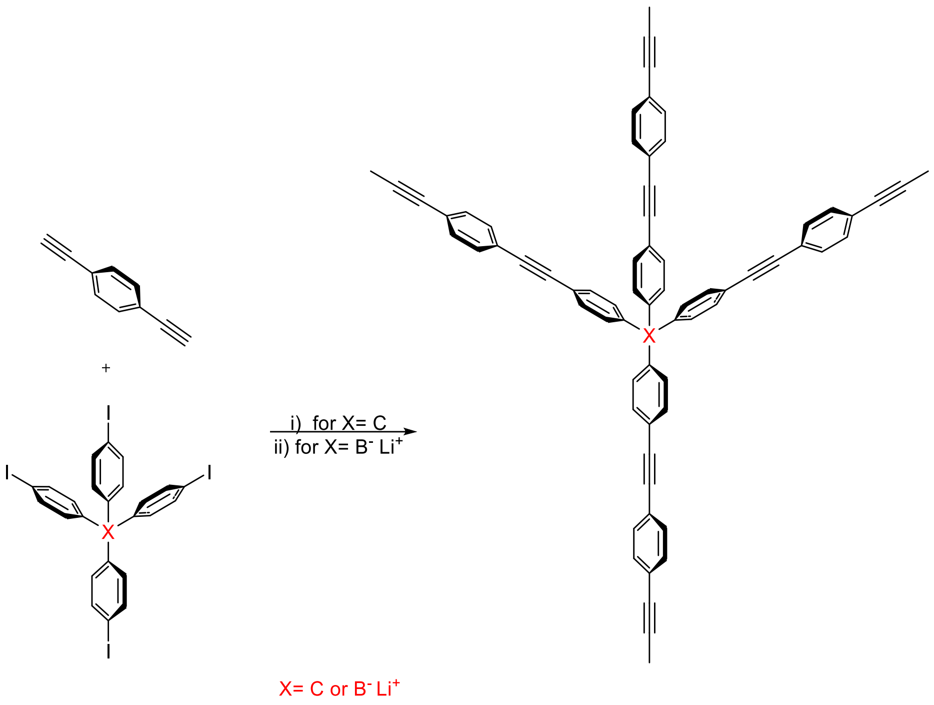

Scheme S2. Synthesis of the framework films using Sonogashira coupling.

Boron-carbon layered COF film synthesis: A quartz crystal microbalance (QCM) Q-Sense Explorer Quartz Crystal Microbalance (QCM-D/ Q-Sense Analyzer) from Biolin Scientific (with Kalrez® O-rings and gaskets) was used. QCM Au sensors containing a Cr adhesive layer were purchased from Biolin Scientific. The Au sensors were cleaned with UV/ozone for two hours and were then immersed in an 80°C 5:1:1 solution of milliQ(mQ)-water, ammonia (32%), and hydrogen peroxide (30%) for 45 min. Next, the cleaned Au chips were washed with mQ-water and EtOH, dried under a N_2 stream, and stored for 1-4 days in a 24-well cell culture plate in a solution of 4-bromothiophenol (1 mM in EtOH). Before film synthesis, the coated sensor was taken out, rinsed with EtOH, blow dried under a stream of N_2, and placed into the QCM chamber. When the temperature was stable at 25°C, the whole set-up was flushed with the solvent mixture (diisopropylamine (DIPA):DMF; 1:1) until all air bubbles were pushed out. Five programmable Legato 110 OEM syringe pumps from KD Scientific were connected to a computer and controlled using the Adagio 1.0 software (see Figure S2). The first pump was charged with a syringe containing a solution of the carbon building block (TIPM, 3.2 mg, 4 µmol, in 20 ml DIPA:DMF; 1:1). The second pump was equipped with a syringe holding a solution of the linker 1,4-diethynylbenzene (1.9 mg, 15 µmol, in 20 ml DIPA:DMF; 1:1). The third pump contained a syringe with the boron building block (LTIPB, 3.2 mg, 4 µmol, in 20 ml DIPA:DMF; 1:1). To the fourth pump was a syringe with the coupling reagents tetrakis-(triphenylphosphine)palladium(0) (4 mg, 3.4 µmol) and copper(I)iodide (2 mg, 11 µmol) dissolved in the solvent mixture (THF:DMF 1:1, 36 ml) added. The fifths pump held a syringe with only the solvent mixture (THF:DMF 1:1, 24 ml). The syringe pumps pushed alternating the fluids from syringe two, three and four (boron-COF layer), one, two and four (carbon-COF layer), or only syringe five (wash), with a flow speed of 1 ml hr^{-1} per syringe pump through the system. The reaction time to grow the carbon or boron layer was set to four hours, while the washing period in between them was one hour, the resonance frequency shifts were constantly measured, and the 3rd overtone was used for further analysis. In the end, syringe pumps one to four were switched off and the COF film was washed for 1-3 hours using the fifth syringe pump (1ml hr^{-1}). The sensor was removed and dried under a stream.

Single layered boron- or carbon-COF films: The procedure was the same as described before, but only with four syringes: The first pump was charged with a syringe containing a solution of the carbon building block (TIPM; 3.2 mg, 4 µmol, in 20 ml DIPA:DMF; 1:1) or the boron building block (lithium tetrakis(4-iodophenyl)borate (LTIPB), 3.2 mg, 4 µmol, in 20 ml DIPA:DMF; 1:1). The second pump was equipped with a syringe holding a solution of the linker 1,4-diethynylbenzene (1.9 mg, 15 µmol, in 20 ml DIPA:DMF; 1:1). The third pump contained a syringe with the coupling reagents tetrakis(triphenylphosphine)palladium(0) (4 mg, 4 µmol) and copper(I)iodide (2 mg, 11 µmol) dissolved in the solvent mixture (THF:DMF; 1:1, 36 ml). The fourth pump held a syringe with only the solvent mixture (THF:DMF; 1:1, 24 ml).

Boron-network film by copper-free reaction: The copper-free Sonogashira coupling was conducted as reported literature and adjusted for continuous flow reaction.[3] Cleaning and templating procedures of the sensor and QCM preparations were the same as described before. When the temperature was stable at 55°C, the whole set-up was flushed with the solvent (MeCN) until all air bubbles were pushed out. Four programmable Legato 110 OEM syringe pumps from KD Scientific were connected to a computer and controlled using the Adagio 1.0 software. The first pump was charged with a syringe containing a solution of the boron building block (LTIPB; 25 mg, 30.4 µmol, in 60 ml MeCN). The second syringe pump was equipped with a syringe holding a solution of the linker 1,4-diethynylbenzene (10.5 mg, 83.2 µmol, in 60 ml MeCN). The third pump contained a syringe with the coupling reagents allyl palladium chloride dimer (31 mg, 84.7 µmol), tri-*tert*-butylphosphine (75 mg, 11 µmol) and 1,4-diazabicyclo[2.2.2]octane (DABCO; 150 mg, 1.34 mmol) dissolved in MeCN (60 ml). The fourth pump held a syringe with only MeCN (24 ml). For the film growth, syringe pumps one to three pushed the fluids with a flow speed of 1 ml hr^{-1} per syringe pump through the system, while the resonance frequency shifts were constantly measured and the 3rd overtone was used for further analysis. In the end, syringe pumps one to three were switched off and the network film was washed for 1-3 hours using the fourth syringe pump (1 ml hr^{-1}). The sensor was removed and dried under a stream of nitroge.

Carbon-network film by copper-free reaction: Cleaning and templating procedures of the sensor and QCM preparations were the same as described before. When the temperature was stable at 45°C, the whole set-up was flushed with the solvent mixture (THF:DMF; 1:1) until all air bubbles were pushed out. Four programmable Legato 110 OEM syringe pumps from KD Scientific were connected to a computer and controlled using the Adagio 1.0 software. The first pump was charged with a syringe containing a solution of the carbon building block (TIPM; 19.8 mg, 24.0 µmol, in 60 ml THF:DMF; 1:1). The second syringe pump was equipped with a syringe holding a solution of the linker 1,4-diethynylbenzene (9.3 mg, 73.7 µmol, in 60 ml THF:DMF; 1:1). The third pump contained a syringe with the coupling reagents $Pd_2(dba)_3$ (60 mg, 65.5 µmol), triphenylarsine 240 mg, 78.4 µmol) and 1,4-diazabicyclo[2.2.2]octane (DABCO; 240 mg, 3.21 mmol) dissolved in the solvent mixture (THF:DMF; 1:1, 60 ml). The fourth pump held a syringe with only the solvent mixture (THF:DMF; 1:1, 24 ml). For the film growth, syringe pumps one-three pushed the fluids with a flow speed of 1 ml hr^{-1} per syringe pump through the system, while the resonance frequency shifts were constantly measured and the 3rd overtone was used for further analysis. In the end, syringe pumps one to three were switched off and the network film was washed for 1-3 hours using the fourth syringe pump (1ml hr^{-1}). The sensor was removed and dried under a stream of nitrogen and the set up was air dried.

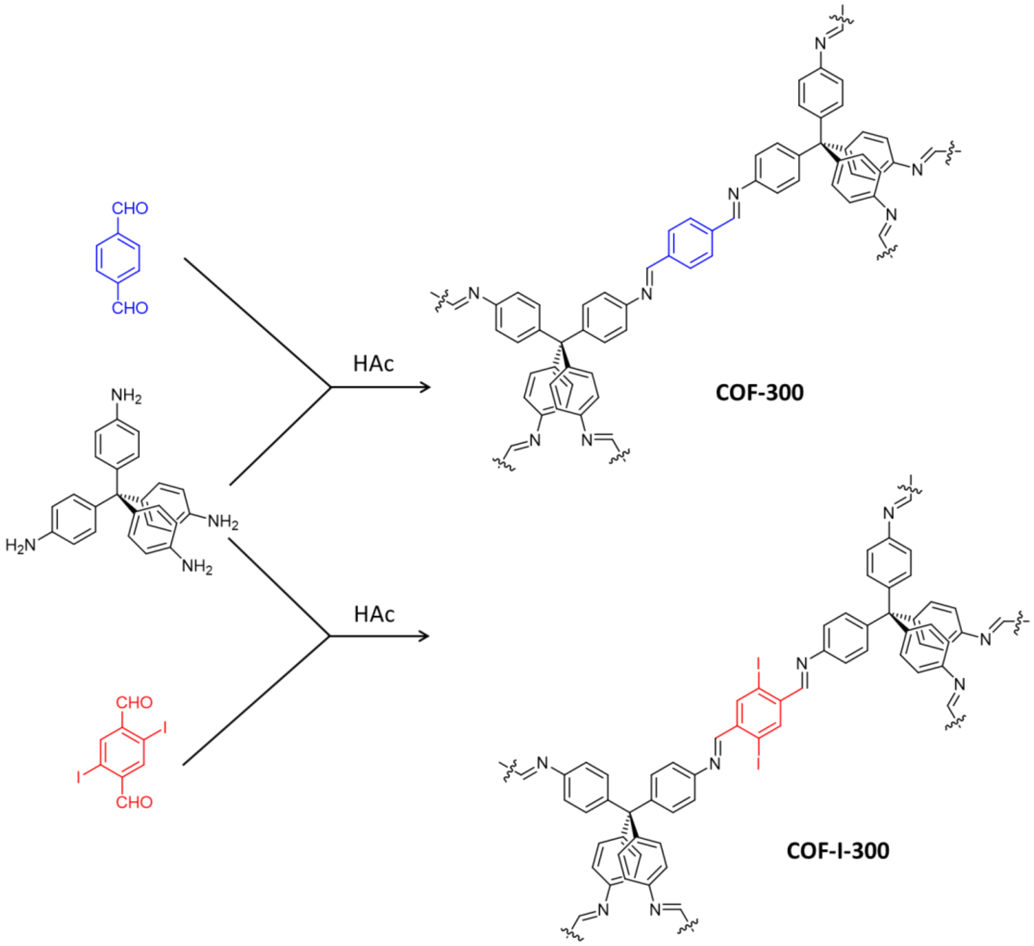

Scheme S3. Synthesis of the imine-type framework films by using Schiff-base chemistry.

The synthesis of COF-300 and COF-I-300 layered film with configuration of A/B/A: The general procedure was the same as the C-C linked layered film preparation. The QCM chips with gold surface were pre-modified by 2-aminothiophenol to form the SAM layer. The temperature of the flow cell was set to 50 ^{o}C. Five syringe pumps were filled with respective tetrakis(4-aminophenyl)methane solution (50 mg, 0.13 mmol, in 12 mL dioxane, syringe 1), terephthalaldehyde solution (30 mg, 0.22 mmol, in 12 mL dioxane, syringe 2), 2,5-diiodoterephthalaldehyde solution (86 mg, 0.22 mmol, in 12 mL dioxane, syringe 3), acetic acid solution (v_{HAc}: $v_{dioxane}$ = 1:4, syringe 4), and pure dioxane (syringe 5).The syringe pumps injected alternating the fluids from syringes 1, 2 and 4 (for COF-300 at the first and the third stage), 1, 3 and 4 (for COF-I-300 at the second stage), or only syringe 5 (for wash between stages) with a speed of 0.7 ml hr^{-1}.The 3rd overtone of the QCM crystal was used for analyze the growth process.

Figures and tables

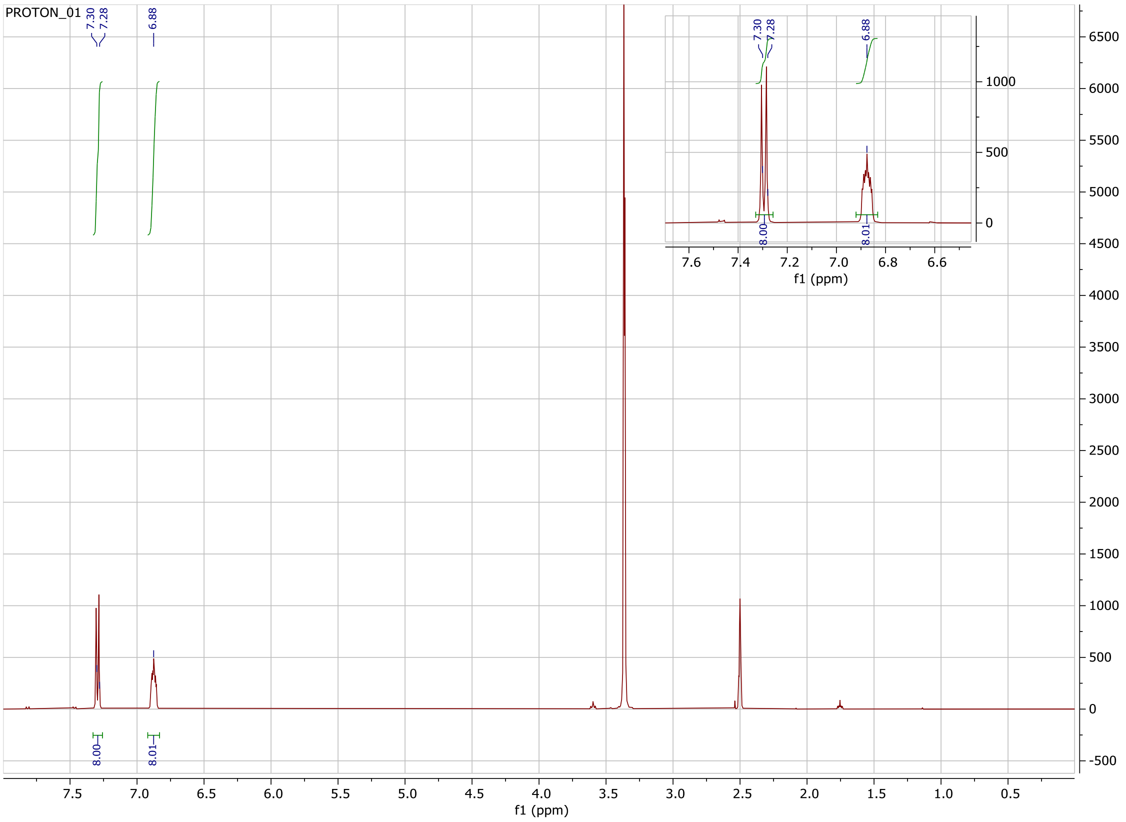

Figure S1. ^{1}H-NMR (400 MHz, dmso-d6) of LTIPB.

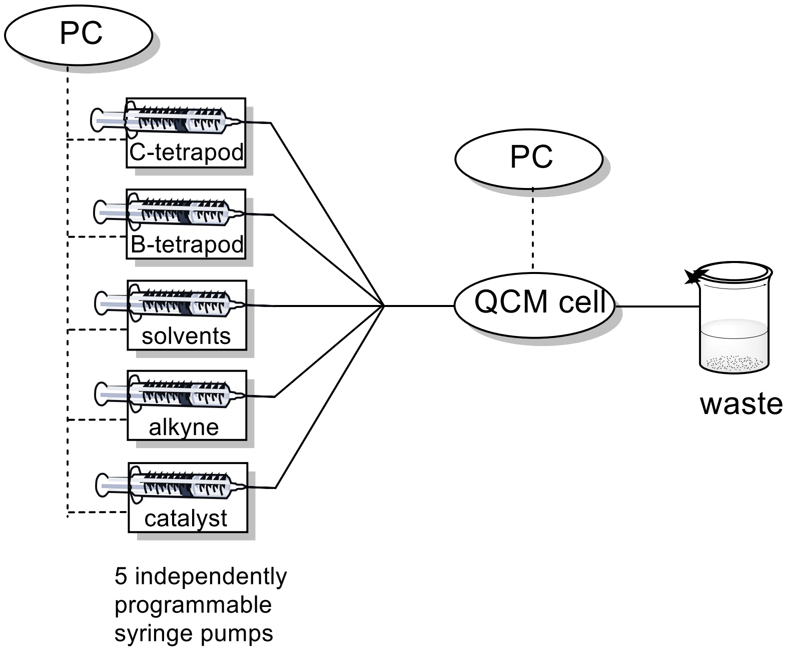

Figure S2. Used set up of syringe pumps and QCM for (layered) COF film growth.

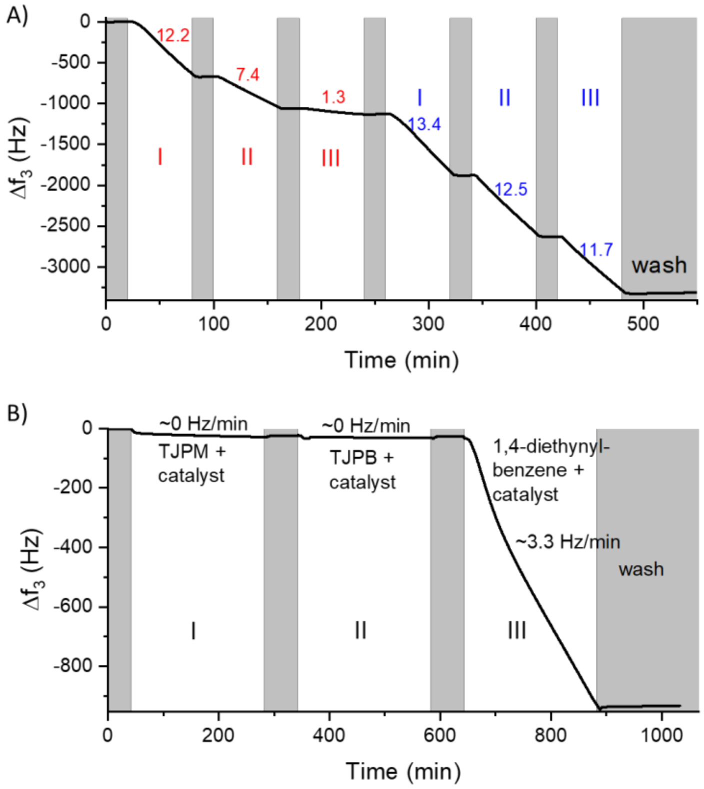

Figure S3. Measured resonance frequency decrease of the quartz crystal during the kinetic study of the coupling reaction. A) In the first area (red I-III) the concentration of the alkyne was kept constant at 7.5×10^{-4} M and the concentration of the TIPM was stepwise reduced. In the second period (blue I-III) the concentration of the TIPM (1.95×10^{-4} M) was kept constant and the amount of the alkyne was reduced. The catalyst concentration (CuI = 2.9×10^{-4} M; $Pd(PPh_3)_4$ = 9.7×10^{-4} M) was kept constant over the entire time of the kinetic study. B) Control experiment showing that no mass adsorption occurs when only flowing TIPM (1.95×10^{-4} M) and catalyst (CuI = 2.9×10^{-4} M; $Pd(PPh_3)_4$ = 9.7×10^{-4} M; black I) or catalyst (CuI = 2.9×10^{-4} M; $Pd(PPh_3)_4$= 9.7×10^{-4} M) and LTIPB (1.95×10^{-4} M; black II) over the surface. In contrast when flowing diethylnylalkyne (7.5×10^{-4} M) together with the catalyst (CuI = 2.9×10^{-4} M; $Pd(PPh_3)_4$ = 9.7×10^{-4} M) mass adsorption on the sensor surface is detectable.

Figure discussion: The kinetic study of the coupling reaction was performed in order to conclude which step in the catalytic cycle that is rate limiting. In Figure S3A the concentration of TIPM (red part) or the alkyne (blue part) was changed while keeping all other concentrations constant. As the concentration of TIPM lowers, the slope indicating the rate of the reaction decreases. On the contrary, when the alkyne concentration lowers, the change of slope is very limited. The alkyne concentration has negligible influence the reaction rate on the surface, showing that the reaction of TIPM in the catalytic cycle is the rate-limiting step for the overall reaction.

Unspecific binding, that is physisorption on the surface, could lead to erroneous conclusions regarding the rate of reactions as the QCM cannot differentiate between materials covalently and physisorbed to the surface. Figure S3B shows the QCM frequency shift when systematically removing one component from the reaction mixture. In the absence of the alkyne the slope of the curve is zero, indicating no reaction nor physisorption of TIPM nor LTIPB on the surface. Interestingly however there was a reaction visible when only the alkyne and catalyst were mixed together. This observation is most likely caused by a Glaser–Hay coupling reaction, in which alkynes homo-couple with each other in the presence of copper and oxygen.[4] The effect of this side reaction on the build-up of the framework is however negligible. This because the rate limiting step in the catalytic cycle involves TIPM, and the catalyst is therefore unable to take part in any side reaction while simultaneously bound to TIPM. This argument is supported by examining the reaction rates in Figure S3A. Firstly, the slope is independent of the alkyne concentration, which would not be the case for a homo-coupling reaction. Secondly, the slope is lower at small TIPM concentrations (Figure S3A red part) than when TIPM is completely absent, which lead to the conclusion that the side reaction was quenched in the presence of TIPM, most likely due to TIPM-catalyst binding.

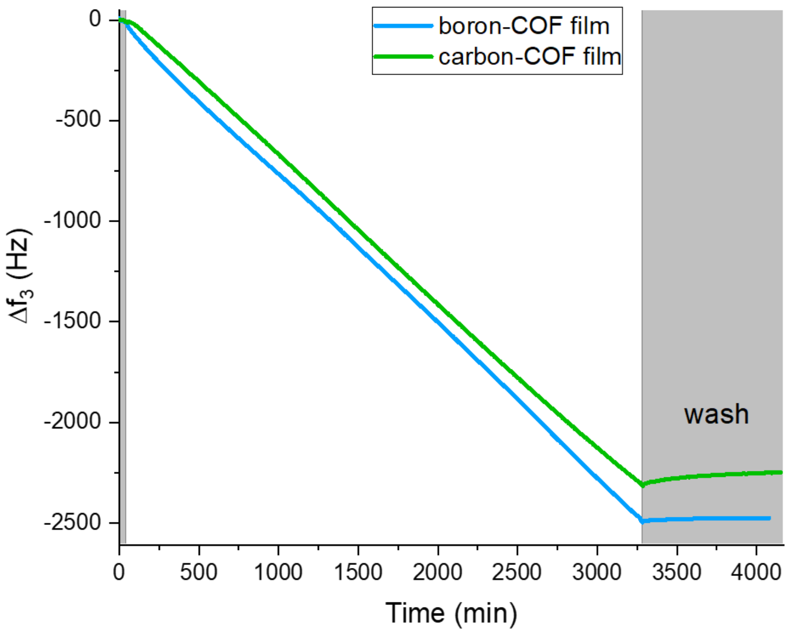

Figure S4. Measured resonance frequency decrease of the quartz crystal during the formation of a B-COF film (green) and a C-COF film (blue) using a copper-free catalytic system.

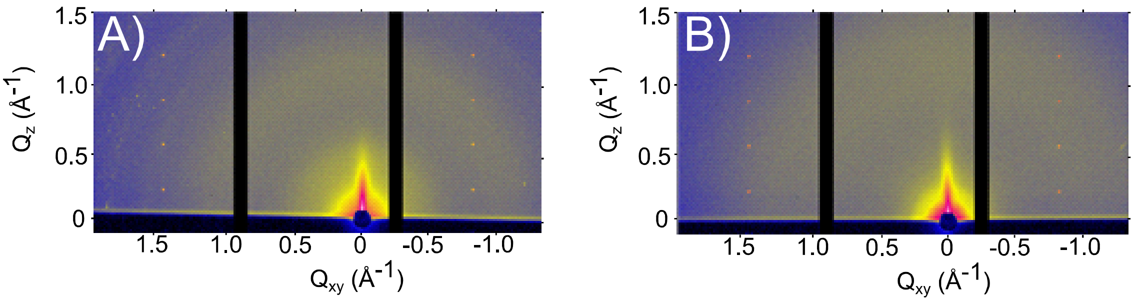

Figure S5. X-ray diffraction pattern (GIXRD) of A) a film synthesized using the boron building block and B) a film synthesized using the carbon building block. Both films were synthesized using the Cu-free catalytic conditions.

Figure discussion: X-ray diffraction showed that the copper-free system doesn't form as crystalline films, as when using conditions involving Cu (**Error! Reference source not found.**). This could be a consequence of the elevated temperatures during the growth of films using the Cu-free system. Since our system is not dynamically bonded like many other COFs, an increased temperature, and with that a higher mobility of molecules, might lead to more disruption in the framework building.

Furthermore, if growing layered films under Cu-free conditions, it is unavoidable to switch between two solvent systems (MeCN for the boron monomer vs. DMF/THF for the carbon monomer). Since the resonance frequency of the QCM chip is calibrated to the first solvent used, the measured resonance frequency would not be stable at solvent changes, preventing direct monitoring of film buildup in real time.

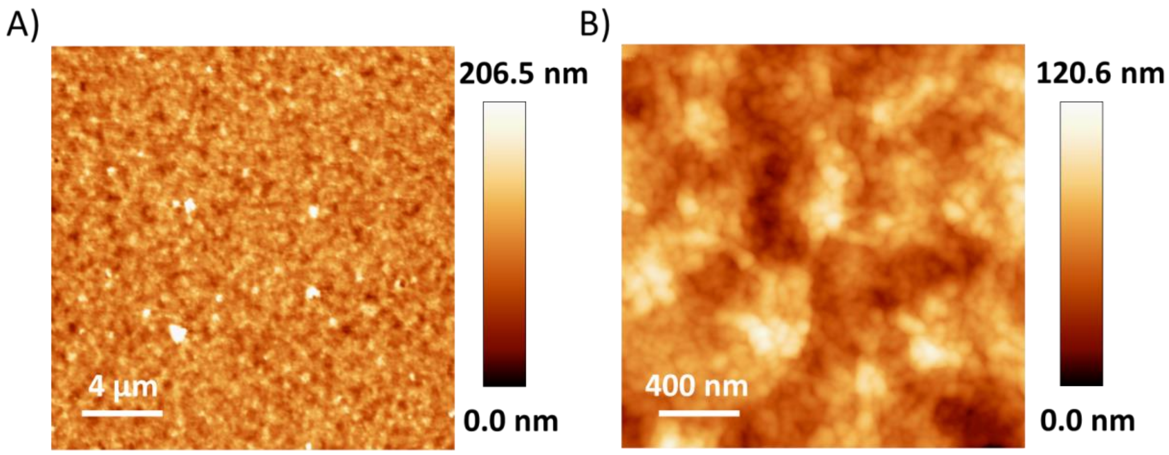

Figure S6. AFM analysis of a boron-COF film. AFM height image of a large surface area A), and a zoom-in area B).

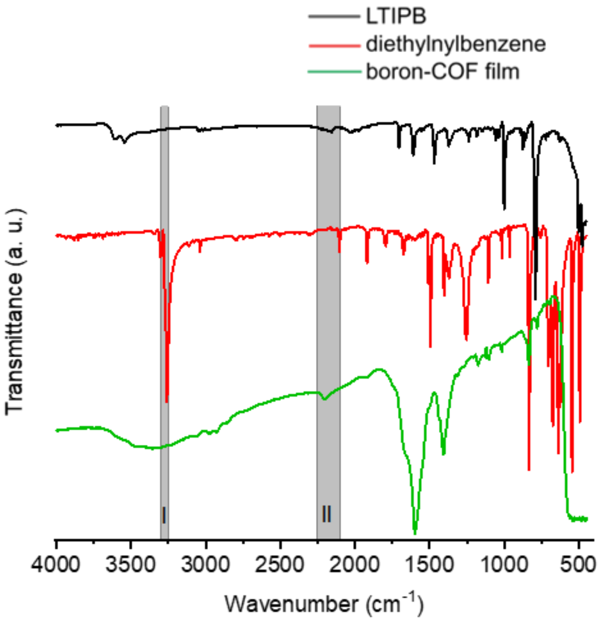

Figure S7. FT-IR of the boron monomer, the alkyne, and a boron-COF film. Region I and II shows the disappearing of reactive groups and forming of new bonds.

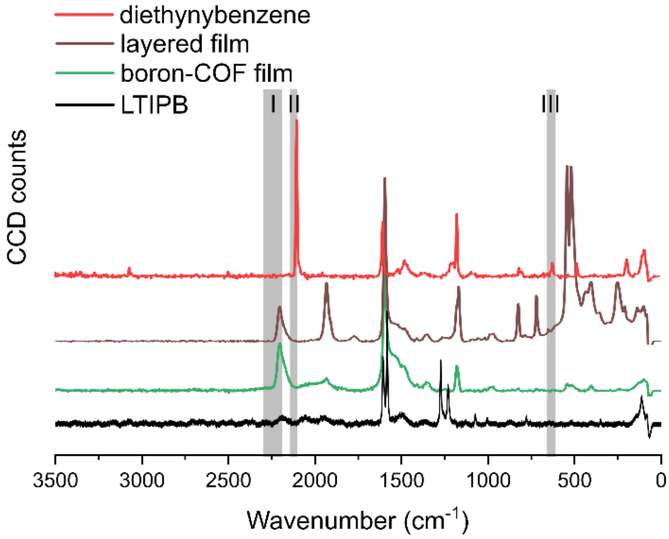

Figure S8. Raman spectra of the boron monomer, the boron-COF film and the layered COF film. Area I marks the expected region for C≡C stretch vibrations of disubstituted alkynes.[5] A signal at 2203 cm^{-1} in the COF films is clearly present, but no signal is evident in the spectra of the building blocks. Area II, between 2140 and 2100 cm^{-1} marks the expected region of C≡C stretch vibrations of terminal alkynes.[5] A strong peak at 2104 cm^{-1} in this region is evident for di-ethynylbenzene, whereas no signal is observed for the COF films.[6] Area III marks the expected region for -C≡C-H bend vibrations. A weak peak in this region is present for diethynylbenzene, but no signal exists for the shown COF films. Thus, similar to Figure 4, there are not many open binding sites in the boron-COF.

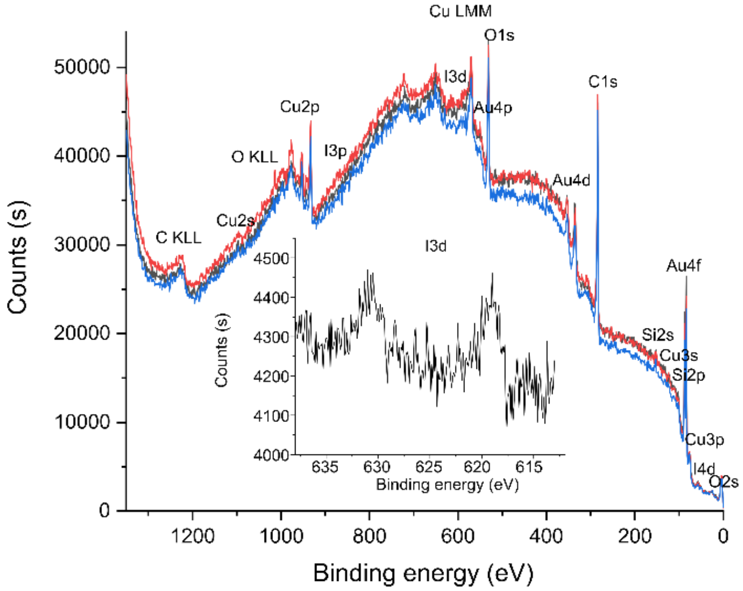

Figure S9. XPS analysis of a carbon-COF film. XPS spectra were measured on three different surface positions for each film (corresponding to red, blue, and black lines). Overview over the whole spectrum, showing the expected signals for carbon, oxygen, silica, copper, iodine and gold. The appearance of gold and silica means that the beam penetrated through the whole film and the results are an average value for the whole film and not just the surface. Inserted is a zoomed in region for the I3d signals from iodine.

Table S1. Elemental composition (atomic percentage) of three different areas on a COF film (resulted from XPS measurements). The small amount of detected iodine indicates a small amount of available binding sites and with that a high degree of polymerization. Perhaps the detected amount origins from the surface, where more binding sites should be present.

	C1s	O1s	Si2s	Cu2p	I3d	Au4f
**area 1**	70.84	20.16	3.00	2.54	0.31	3.16
**area 2**	71.74	19.92	2.06	3.60	0.00	2.68
**area 3**	70.04	20.60	4.86	2.19	0.00	2.31
**average**	70.87	20.23	3.31	2.77	0.10	2.71
**standard deviation**	0.85	0.34	1.43	0.73	0.18	0.43

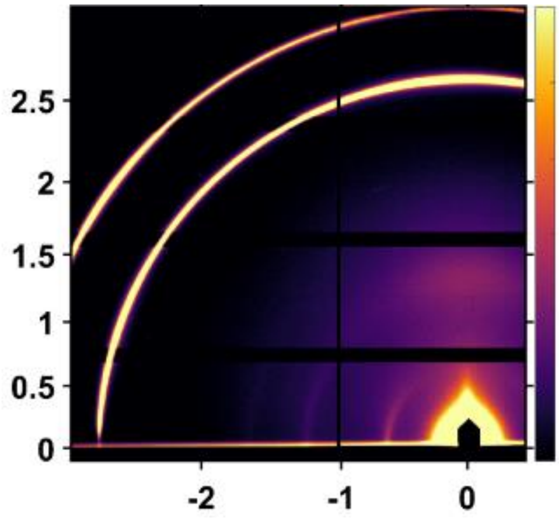

Figure S10. GIXRD measurement at Argonne National Laboratory, Sector 8, of a carbon-COF film, which was made in an identical Sonogashira coupling, with tetrakis(4-bromophenyl)methane (TBPM) as building block instead of the iodine-analogue. The diffraction pattern looks the same as for the frameworks which were synthesized using tetra(4-iodophenyl)methane and/or lithium tetrakis(4-iodophenyl)borate.

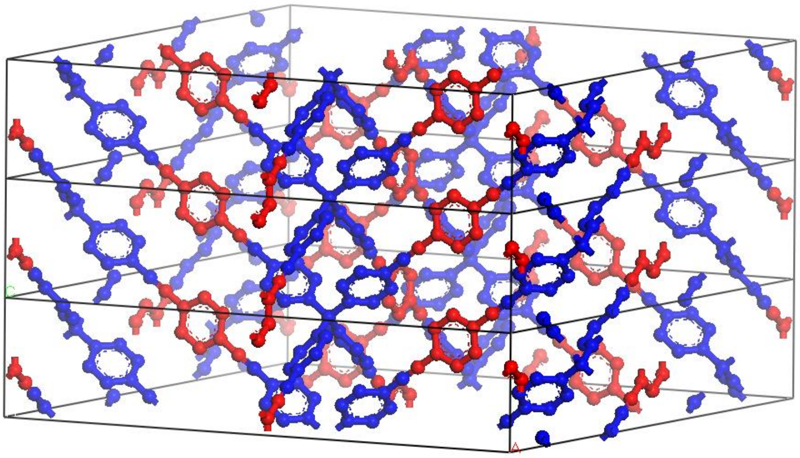

Figure S11. The simulated framework structure for carbon- and boron-COF. The red part of the structure highlights the linear linker (dietyhnylbenzene), whereas the tetrahedral TIPM/LTIPB is shown in blue. The unit cell dimensions are a=b=31.8 Å, c=6.6 Å; alpha=beta=gamma=90°.

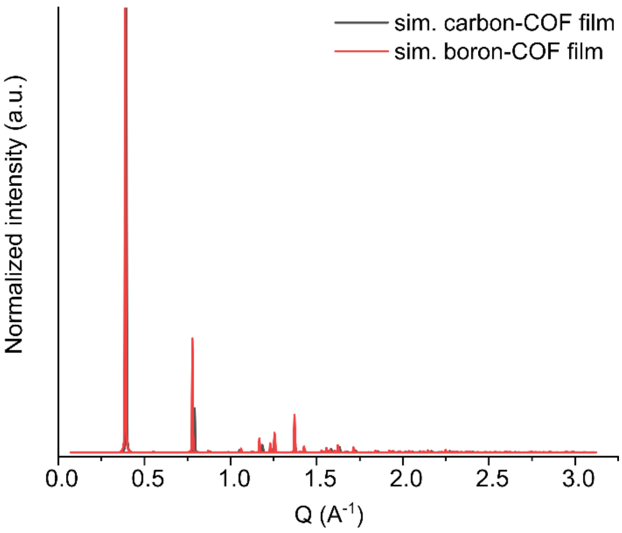

Figure S12. Comparison of the simulated diffraction signal from the boron- and the carbon-COF films. The graph confirms the similarity in between the simulated diffraction peaks for the carbon- and the boron-COF. In this scattering model, the borate counterion is ignored, as it is assumed to be randomly dispersed in the chemical network.

Figure discussion: Crystal modeling of the non-interpenetrated 3D lattice was carried out using Accelrys Materials Studio (ver.5.0). Initial structures were constructed by first estimating the primitive symmetry (a diamonoid network) and known bond lengths. Backgrounds were removed via the Reflex module with a polynomial approximation with 30 iterations and an averaging window size of 0.9°. These structures were then optimized using a Geometry Optimization routine including energy minimization with cell parameters optimization, using the parameters from the Universal Force Field. Calculation of the simulated powder diffraction patterns and Pawley refinements were performed in the Materials Studio Reflex Plus Module using a Bragg-Brentano geometry with copper Kα radiation. Importantly, it was not possible to observe low scattering features due to experimental limitations with our GI-XRD setup. This obfuscates our ability to assign the interpentration of the network reliably. Nonetheless, the structure observed here agrees well with experimentally observed higher order diffraction features, which indicates that the parent diamond framework is a good representation of the unambigously crystalline material.

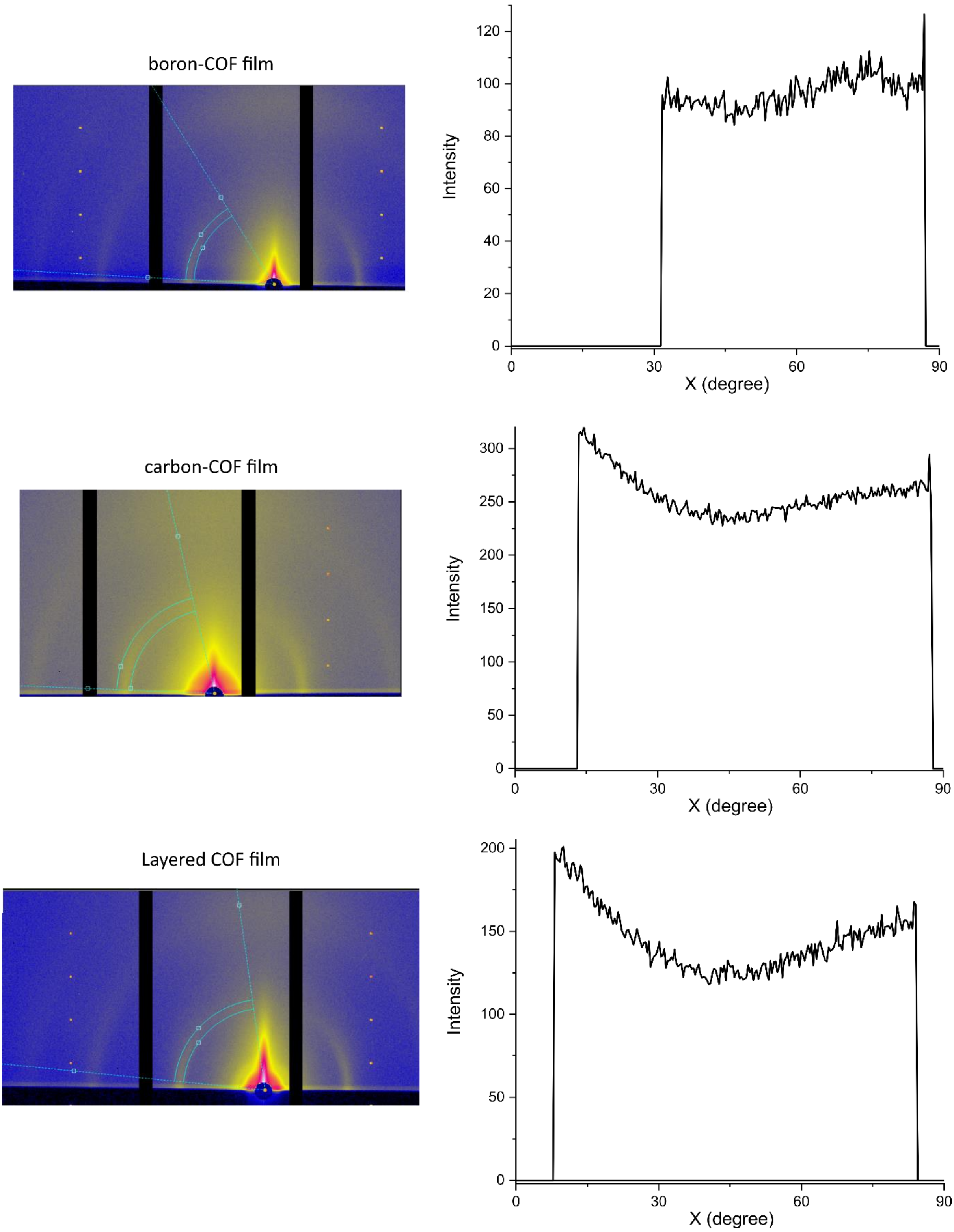

Figure S13. Integration of the half-spherical diffraction intensity for boron-, carbon- and layered COF films. The intensity in all three spectra increases towards the horizon (x = 90), which indicates a preferred orientation of the framework.

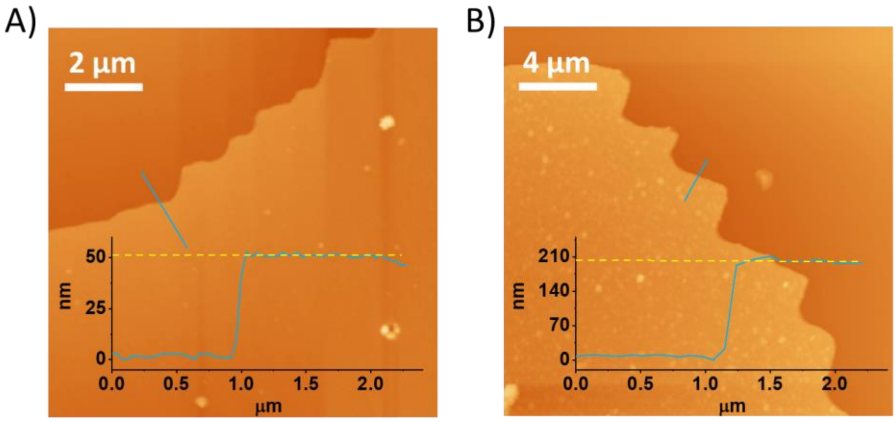

Figure S14. A) AFM heigh image of one layer carbon-COF film (f = 1290 Hz), having a thickness of 52 nm. B) AFM heigh image of a four-layer alternating carbon-boron COF film (f = 4885 Hz) having a thickness of 203 nm. The insets show the extracted sectional profile along the edge of the scratched film.

Figure discussion: By combing the measured thickness of the film with the QCM data, the density of the film can be calculated. According to the Sauerbrey equation: $\Delta m = -C \cdot \frac{\Delta f}{n}$

where:

C = 4.4 ng/(cm^2 Hz) for the used 10 MHz quartz crystal,

Δf = -1221 Hz, which is the average frequency decrease for each layer,

n = 3, the data from the third harmonic was used applied.

The mass accumulation for each growth stage is then Δm = 1791 ng/cm^2

The density of the film can then be calculated: $\rho = \frac{\Delta m}{V} = \frac{1791\ ng}{52\ nm \times 1\ cm^2} = 0.34$ g/cm^3

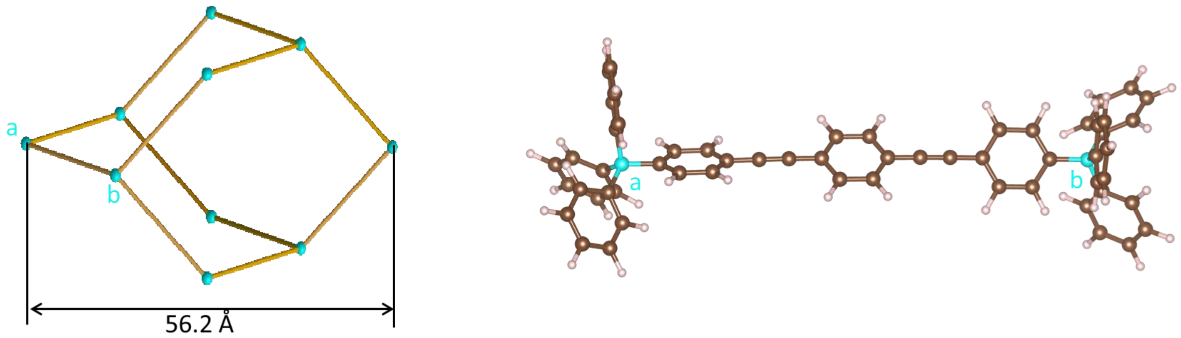

Figure S15. The simulated size of a unit cage with diamond topology. According to the simulation, the size of a unit cage in the structure is 56.2 Å. The thickness of each layer is about 52 nm, as measured by AFM. Thus, each growth stage enables the build-up of 52/5.62 ≈9 unit cages.

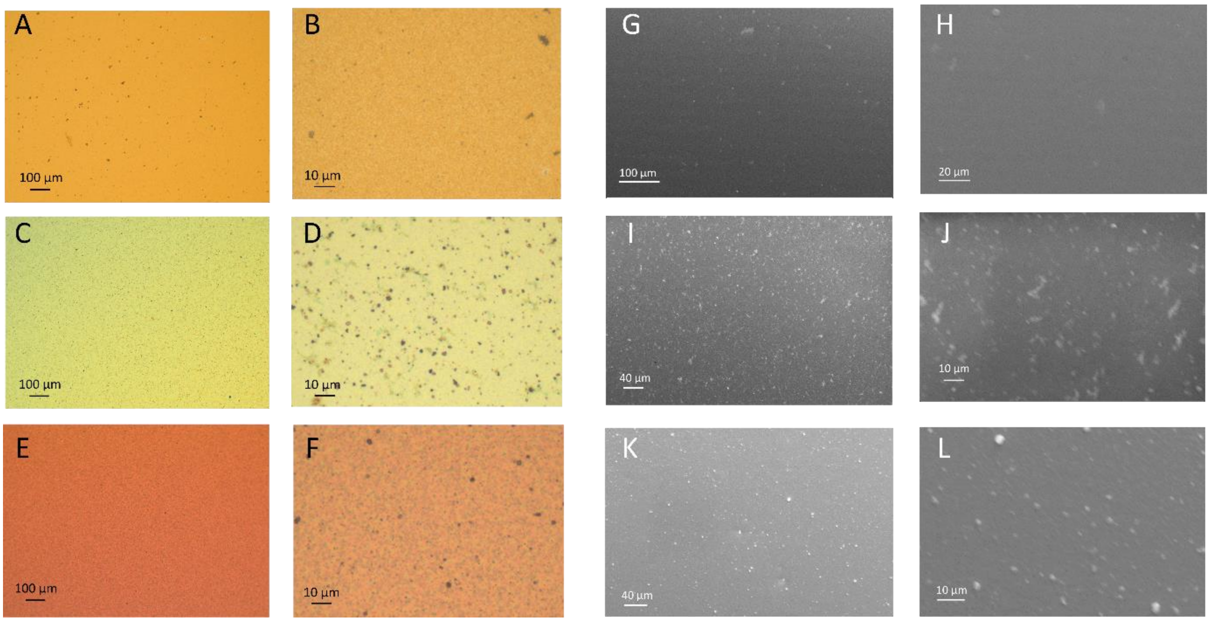

Figure S16. Optical microscope and SEM images of film surfaces at different magnifications. The images in the first row are taken from a carbon-COF film, the middle row shows the surface of a boron-COF film and the lower pictures show a layered COF film.

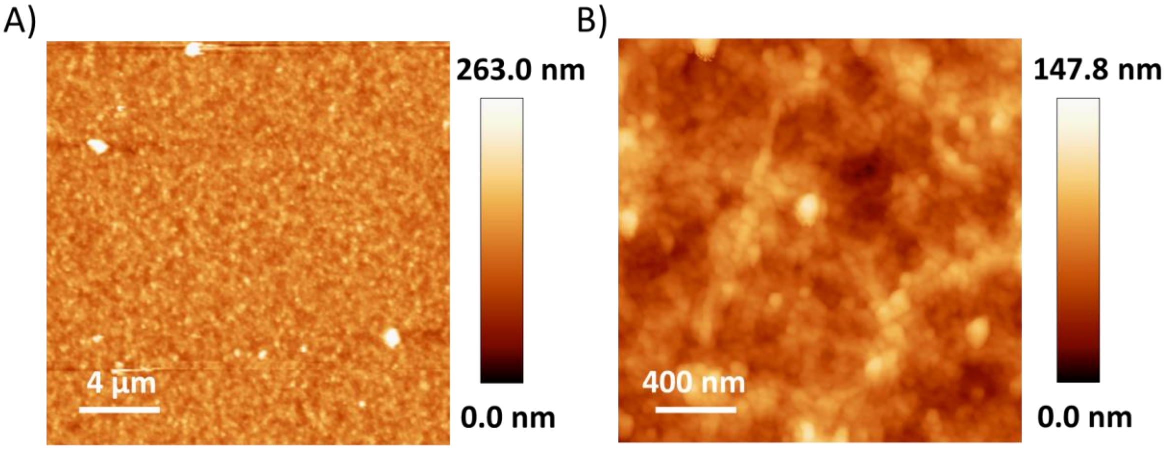

Figure S17. AFM height image of layered COF film A) and a zoom-in scan for surface morphology in higher magnification B).

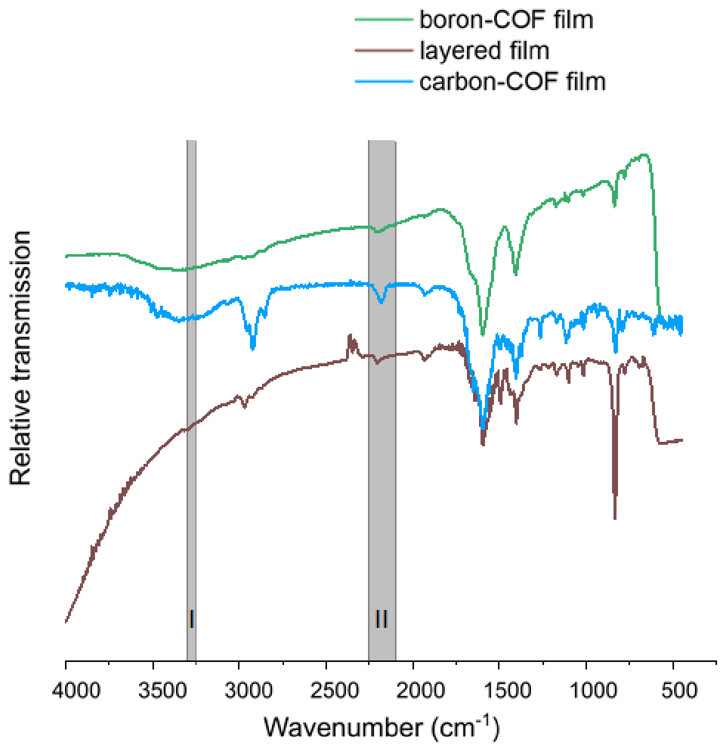

Figure S18. Comparison of FT-IR spectra of layered COF film with single component of boron-COF and carbon-COF.

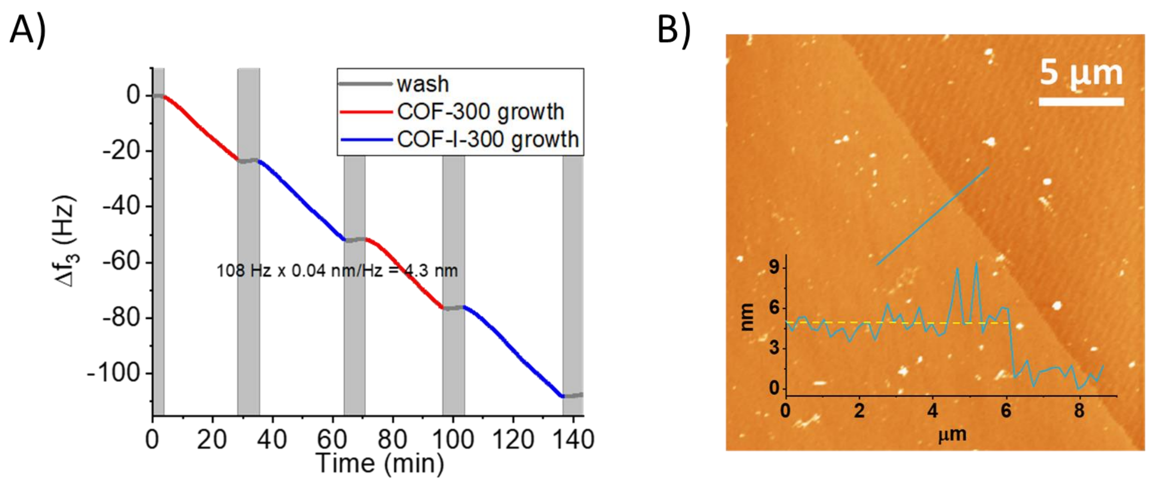

Figure S19. Frequency decrease of the quartz crystal monitoring the periodical growth of ultra-thin COF-300 and COF-I-300 alternating layers A) and the measured thickness B) by AFM.

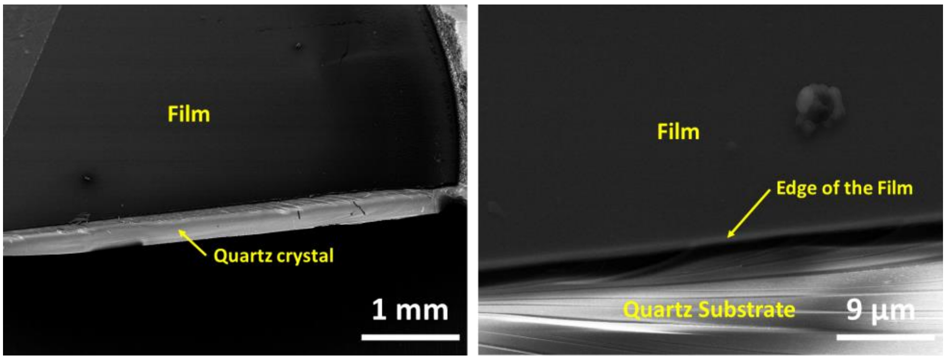

Figure S20. SEM images of a fractured COF film on the quartz crystal substrate. The film has the structure of alternating COF-300 and COF-I-300 layers in A/B/A configuration.

References

(1) Malek, N.; Maris, T.; Simard, M.; Wuest, J. D. Molecular Tectonics. Selective Exchange of Cations in Porous Anionic Hydrogen-Bonded Networks Built from Derivatives of Tetraphenylborate. J. Am. Chem. Soc. 2005, 127 (16), 5910-5916. DOI: 10.1021/ja042233m.

(2) Ma, T.; Kapustin, E. A.; Yin, S. X.; Liang, L.; Zhou, Z.; Niu, J.; Li, L.-H.; Wang, Y.; Su, J.; Li, J.; et al. Single-crystal x-ray diffraction structures of covalent organic frameworks. Science 2018, 361 (6397), 48-52. DOI: doi:10.1126/science.aat7679.

(3) Soheili, A.; Albaneze-Walker, J.; Murry, J. A.; Dormer, P. G.; Hughes, D. L. Efficient and General Protocol for the Copper-Free Sonogashira Coupling of Aryl Bromides at Room Temperature. Org. Lett. 2003, 5 (22), 4191-4194. DOI: 10.1021/ol035632f.

(4) Akhtar, R.; Zahoor, A. F. Transition metal catalyzed Glaser and Glaser-Hay coupling reactions: Scope, classical/green methodologies and synthetic applications. Synth. Commun. 2020, 50 (22), 3337-3368. DOI: 10.1080/00397911.2020.1802757.

(5) Graham, L. J. B. S. H. F. L. D. A. C. R. Organic structural spectroscopy; Prentice-Hall, Inc, 2001.

(6) Lim, J. K.; Joo, S.-W.; Shin, K. S. Concentration dependent Raman study of 1,4-diethynylbenzene on gold nanoparticle surfaces. Vib. Spectrosc. 2007, 43 (2), 330-334. DOI: https://doi.org/10.1016/j.vibspec.2006.04.006.
